# Successful Use of Extracorporeal Life Support and Continuous Renal Replacement Therapy in the Treatment of Cardiogenic Shock Induced by Tumor Lysis Syndrome in a Pediatric Patient With Lymphoma: A Case Report

**DOI:** 10.3389/fmed.2021.762788

**Published:** 2022-01-04

**Authors:** Zhulin Wang, Fang Zhang, Long Xiang, Yinyu Yang, Wei Wang, Biru Li, Hong Ren

**Affiliations:** ^1^Department of Pediatric Intensive Care Unit, Shanghai Children's Medical Center, Shanghai Jiaotong University School of Medicine, Shanghai, China; ^2^Department of Pediatric Thoracic and Cardiovascular Surgery, Shanghai Children's Medical Center, Shanghai Jiaotong University School of Medicine, Shanghai, China

**Keywords:** ECMO (extracorporeal membrane oxygenation), tumor lysis syndrome, continuous renal replacement therapy (CRRT), pediatric, case report

## Abstract

The use of extracorporeal membrane oxygenation (ECMO) in the treatment of cardiopulmonary failure in children with malignant tumors is controversial. There are few reports on the use of ECMO in the treatment of children with tumor lysis syndrome. This article reports a case of a 9-year-old girl who presented with hyperkalemia and cardiogenic shock. The discovery of an abdominal mass with critical ultrasound provided key evidence for the initial diagnosis of tumor lysis syndrome. Cardiopulmonary resuscitation was performed for 1 h. Veno-arterial ECMO was installed at the bedside to provide cardiopulmonary support for the patient and was combined with continuous renal replacement therapy (CRRT) to improve her internal environment. The patient was ultimately diagnosed with mature B-cell lymphoma with tumor lysis syndrome. A severe electrolyte disorder led to cardiogenic shock. After the electrolyte imbalance was corrected, the patient's heart function gradually improved, ECMO was successfully weaned, and chemotherapy was continued with the support of CRRT. One month after ECMO weaning, the organ function of the patient had recovered and there were no serious complications. In this case report, we paid attention to the rapid diagnosis of the etiology behind a patient's shock with critical ultrasound as well as the initiation and management of extracorporeal cardiopulmonary resuscitation (ECPR), which provided us with valuable experience using VA-ECMO on critically ill children with tumors. It is also important evidence for the use of ECMO in the treatment of children with cardiopulmonary arrest secondary to malignancy.

## Introduction

Extracorporeal cardiopulmonary resuscitation (ECPR) can greatly improve the survival rate of both in-hospital and out-of-hospital cardiac arrest. Early diagnosis of the cause of cardiogenic shock can greatly improve treatment efficiency, shorten time on extracorporeal membrane oxygenation (ECMO), and reduce the economic cost of medical care. Bedside critical ultrasound can guide the diagnosis and treatment of patients in cardiogenic shock and is also an important bedside assessment method for guiding the initiation of ECPR.

Tumor lysis syndrome (TLS) is a critical complication of malignancy that frequently occurs in children with leukemia and lymphoma. TLS is characterized by internal environmental disorders such as hyperkalemia, hyperphosphatemia, hypocalcemia, and hyperuricemia, which cause renal failure and severe cardiac arrhythmias (1). ECMO has been shown to benefit children in severe respiratory or cardiac failure, but its use in patients with malignancies is still controversial, especially in children (2). The chief concerns regarding ECMO in patients with cancer focus on the poor prognosis of malignancy and the serious complications of ECMO, such as infection and bleeding. As the long-term prognosis of most childhood tumors has greatly improved, more medical centers have begun to explore the beneficial effects of ECMO in children with malignancies. However, the timing and contraindications of ECMO in children with tumors still require further clinical exploration (3).

In this study, we report the case of a 9-year-old girl who was admitted to the pediatric intensive care unit (PICU) in shock with ventricular arrhythmia. We rapidly diagnosed the cause of her cardiogenic shock and TLS within 30 min of admission based on cardiopulmonary ultrasound findings, the discovery of an abdominal mass, and internal environmental characteristics such as hyperkalemia and hypocalcemia. Extracorporeal cardiopulmonary resuscitation (ECPR) was performed at the bedside after an additional 30 min, and the child received ECMO + continuous renal replacement therapy (CRRT) for 3 days. The child was successfully weaned off ECMO and discharged after completing chemotherapy.

## Case Report

### Patient Information

A 9-year-old girl with a history of “arthritis” over the prior 2 months was admitted to the pediatric intensive care unit (PICU) for 1 day of abdominal distension and 30 min of dyspnea. An electrocardiogram showed short bursts of ventricular tachycardia. Her initial diagnosis in the outpatient clinic was “fulminant myocarditis, ventricular arrhythmia, and cardiogenic shock (compensated period).” The child took 10 mg of methotrexate for “arthritis” 1 day ago and denied any other past medical history.

### Clinical Findings

Physical examination suggested that the child was in the compensatory period of shock: she was irritable, dyspneic with 86% transcutaneous oxygen saturation under mask oxygen, her heart rate was 120 beats/min, her blood pressure was 70/42 mmHg, and her capillary refill time was 4–5 s. Other positive physical examination findings included: fine wet rales in her lungs, reduced heart sounds, and a suspicious mass in her lower abdomen.

### Diagnostic Assessment

**Figure 1:**
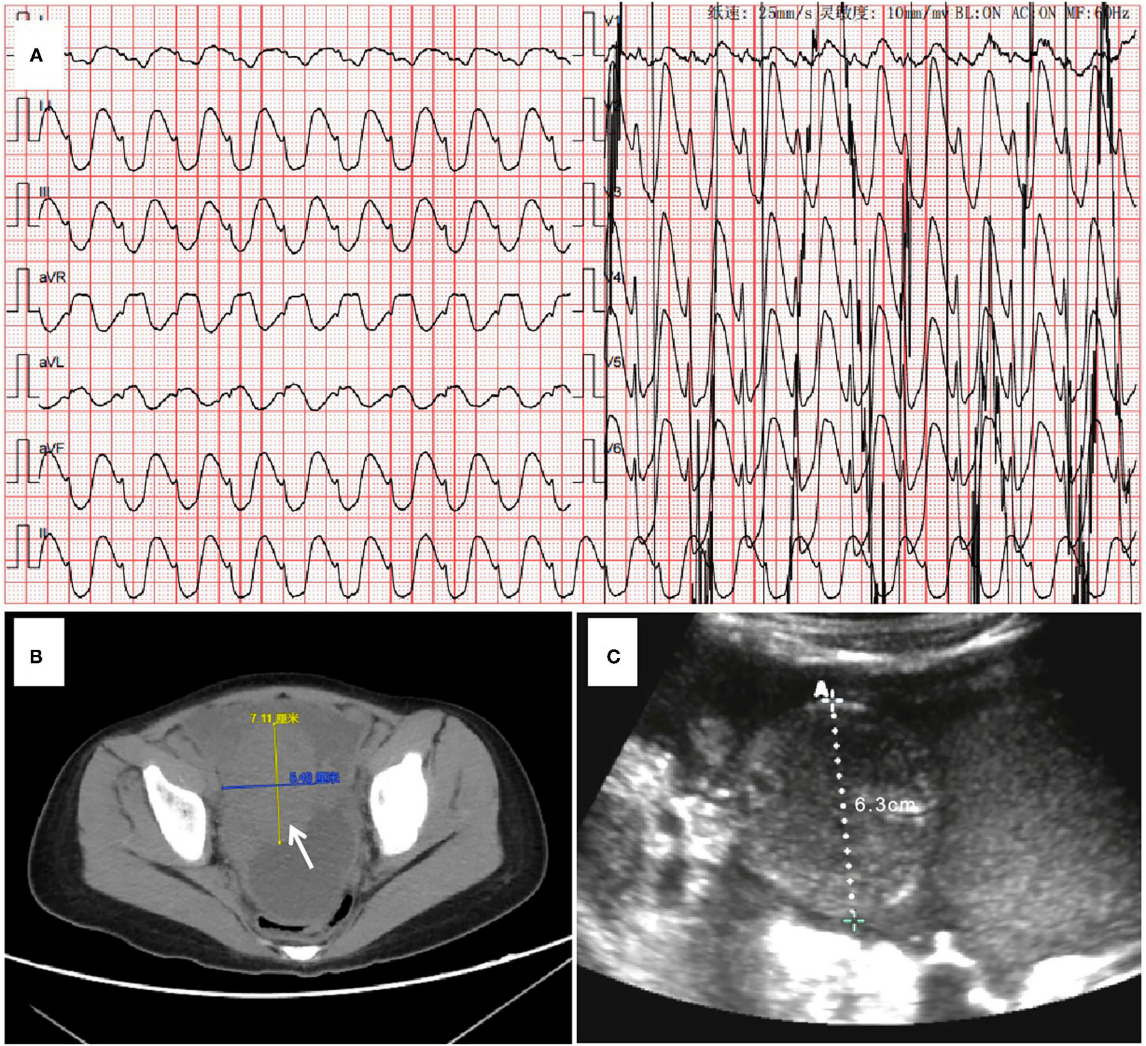
Sequential imaging evaluations performed on Day 0. **(A)** The electrocardiogram on admission indicates ventricular tachycardia. **(B)** The abdominal CT indicates the presence of space. **(C)** The rapid bedside abdominal B-ultrasound prompts abdominal occupation.

The patient's bedside electrocardiogram showed a ventricular arrhythmia (Figure 1A), and her blood gas analysis showed hyperkalemia, hypocalcemia, and metabolic acidosis (Table 1). After admission, the child had repeated episodes of pulseless ventricular tachycardia requiring bedside conventional cardiopulmonary resuscitation (CCPR). A tracheal intubation ventilator was used to support breathing. Intravenous drugs (calcium, insulin, adrenaline) were immediately used. An emergency bedside ultrasound revealed a significant decrease in the patient's left ventricular systolic function, with a velocity–time integral of 2 cm, a left ventricular ejection fraction (LVEF) of 15%, volume overload (enlargement of the right ventricle), and a right pleural effusion. However, the results of the outpatient blood test showed cardiac troponin (c-TNI) 0.06 μg/L and CK-MB(m) 1.90 μg/L, which are not in line with the common characteristics of fulminant myocarditis. The cause of the patient's cardiogenic shock was therefore difficult to diagnose: was it really cardiogenic shock caused by fulminant myocarditis that led to an imbalance of the patient's homeostasis? Could an alternative reason result in internal environmental imbalance (low calcium, high potassium) and then cause cardiogenic shock? A bedside abdominal ultrasound at the bedside provided a key piece of evidence: an abdominal pelvic effusion with a substantial mass in the pelvic cavity (Figure 1C). The patient's outpatient computed tomography (CT) also showed pleural effusion and a pelvic space mass (Figure 1B), but due to the emergency situation, her CT images were not uploaded to the hospital imaging network on time. The patient's preliminary diagnosis was therefore “pelvic neoplasm, TLS, hyperkalemia, ventricular arrhythmia, and cardiogenic shock.” On the second hospital day, a diagnosis of mature B-cell lymphoma (stage IV, R4) was confirmed *via* flow cytometry of the ascites.

**Table 1:** Hemodynamic and laboratory measurements over the course of the ECMO treatment of the patient.

**Time after ICU admission**	**Day 0**	**Day 1**	**Day 2**	**Day 3**	**Day 4**
	**Peri-ECMO**	**Under-ECMO**	**Under-ECMO**	**ECMO weaning**	**Post-ECMO**
**Hemodynamic parameters**					
ECMO Flow (L/min)	2.79	2.39	2.4	1.5	-
Blood pressure (mmHg)	72/60	79/61	71/60	95/62	110/55
CVP (cm H2O)	8	12	9	8	12
ScvO2 (%)	75	63	66	69	55
PCO2 gap	10	11	10	4.3	7.1
LVOT-VTI (cm)	2	4	8	13	14
Fluid balance (ml)	+2,854	+640	+550	+194	−11
Adrenaline (μg/kg/min)	0.5	0.4	0.3	0.25	0.05
Norepinephrine (μg/kg/min)	0.5	0.2	0	0	0
**Homeostasis parameters**					
PH	6.72	7.36	7.33	7.39	7.4
PaO2 (mmHg)	81.3	118	115	210	99.1
PaCO2 (mmHg)	53.2	38.7	39.1	38.5	38.8
Lac (mmol/L)	26	12	4.9	1.2	1
K+ (mmol/L)	9.03	6.11	3.98	4	3.47
Free Ca2+ (mmol/L)	1.3	1.78	1.8	2.16	2.1
Phosphorus (mmol/L)	8.4	3.98	1.38	1.01	0.66
Uric acid (μmol/L)	2,620	1,198	471	-	85.5
Creatinine (μmol/L)	177	73	42	-	48

*CVP, central venous pressure; ScvO2, central venous oxygen saturation; PCO2 gap, central venous to arterial carbon dioxide partial pressure difference; LVOT-VTI, left ventricular outflow tract velocity time integral; PH, Potential of Hydrogen; Lac, lactic acid*.

### Therapeutic Intervention

Within 30 min of admission, the child had experienced four episodes of pulseless ventricular tachycardia and required three electrical cardioversions. Spontaneous circulation was restored 2–4 min after CCPR after the first three arrests. During the fourth episode of ventricular fibrillation, EPCR was initiated. Continuous advanced CPR was performed for another 40 min under invasive arterial blood pressure monitoring (MAP > 60 mmHg). Veno-arterial ECMO (VA-ECMO) was then performed *via* peripheral cannulation of the right femoral artery using 15 Fr arterial cannulae [Medtronic], a 19 Fr right femoral vein cannula catheter [Medtronic], and a distal perfusion tube 6 Fr [Tyrmer], which took the surgeon 40 min. The ECMO circuit was previously circulated with Ringer acetate (500 ml), 20% albumin (50 ml), and heparin (10 mg). A red blood cell suspension (1 U) was then pre-filled throughout the whole line to ensure hematocrit > 30%. A Maquet (Rastatt; Baden-Württemberg, Germany) PLS Membrane and Rotaflow centrifugal Pump (Maquet Cardiopulmonary AG, Hirrlingen, Germany) were utilized. Support was initiated using 90 mL/kg/h pump flow, 2,100 revolutions per minute (RPM) at a 3,000 mL/min sweep, and a 50% fraction of inspired oxygen. Considering that the cause of the patient's cardiogenic shock was an internal environmental disturbance such as hyperkalemia induced by TLS, CRRT is synchronized to prepare and pre-charge for an operation to correct the internal environmental disturbances and replace the kidneys to reduce capacity load. CRRT was connected to the ECMO circuit, with the access line connected after the oxygenator and the return line connected before the oxygenator. A CVVHDF mode was adopted. Blood flow speed was 250 ml/min (6.25 ml/kg/min), dialysate speed was 2,000 ml/h (50 ml/kg/h), the replacement fluid speed was 1,000 ml/h (25 ml/kg/h), and the dehydration speed was adjusted over time. During the ECMO maintenance, the fluid balance was mainly kept positive to preserve ECMO flow and blood pressure. The patient's condition improved significantly 4 days after admission, and reverse fluid resuscitation began to be achieved (Table 1). The ventilator was set to PC mode, with peep 5 cm H2O, a peak inspiratory pressure (PIP) of 18 cm H2O, a tidal volume (VT) of ~200 ml (4–5 ml/kg), a respiratory rate (RR) of 13 times/min, and a FiO2 (fraction of inspired oxygen) of 80%. Considering the higher risk of bleeding in the patient, we adopted a conservative anticoagulation strategy of whole-body heparinization with heparin sodium 5–10 IU/kg/h for intravenous maintenance. The patient's activated clotting time was ~180 s and her activated partial thromboplastin time was ~60 s (Table 2). Since the total CCPR time was up to 1 h, to avoid secondary damage to the brain the following measures were taken: (1) control the child's temperature at 35°C through the ECMO device within 4 h after running. After a total of 2 days of mild hypothermia treatment, the day before ECMO weaning the patient was slowly rewarmed at 0.2°C/h until her body temperature was 36.5°C; and (2) combined use of sedative and analgesic drugs and muscle relaxants to maintain a deep sedative and analgesic level with a Richmond Agitation—Sedation Scale (RASS) of −4 to −5 points and a bispectral index (BIS) of 40–50; (3) cerebral blood flow signals monitored by transcranial doppler to preserve the time-average flow velocity of the middle cerebral artery at 70–100 cm/s by adjusting vasoactive drugs or ECMO flow. Local cerebral oxygen saturation (nirs-sco2) was maintained at more than 60%. As ECMO catheterization is an invasive operation, vancomycin combined with ceftazidime was used intravenously for infection prophylaxis. On the third day of ECMO, the patient's C-reactive protein (CRP) and procalcitonin levels increased significantly. Antibiotics were therefore changed to vancomycin combined with meropenem to fight the infection. With the use of tumor chemotherapy drugs, the patient's white blood cell count was reduced to 0.5^*^10^∧^9/L but her CRP remained high 6 days after admission. Caspofungin was therefore added as empiric antifungal treatment.

**Table 2:** Additional biologic parameters, treatments, and relevant data during the ECMO treatment.

**Timeline after ICU admission**	**Day 0**	**Day 1**	**Day 2**	**Day 3**	**Day 4**
	**Peri-ECMO**	**Under-ECMO**	**Under-ECMO**	**ECMO weaning**	**Post-ECMO**
**Coagulation parameters**					
Heparin (UI/kg/h)	5	7.5	10	7.5	0
ACT (S)	220	190	204	180	-
APTT (S)	69	45	69	67	36
Anti-X (IU/ml)	0.03	0.01	0.17	0.16	-
AT (%)	-	33	-	27	-
Platelet(x10^∧^9/L)	103	88	21	13	18
**Infection and inflammation parameters**					
CRP (mg/L)	73	53	120	140	106
Procalcitonin (ng/ml)	1.27	-	-	75	-
White blood cell (x10^∧^9/L)	20.6	10.6	1.38	1.15	0.99
Neutrophil (%)	39	30.7	55	43	39
Lymphocyte (%)	53	64.5	39	50.4	53.5
Monocyte (%)	5.9	2.6	2.2	1.7	3
Ferritin(ng/ml)	-	314	-	6,000	-
Interleukin-6 (pg/ml)	-	-	-	5,378	-
Etiology	Sputum/Blood/Urine Culture (-)				
Antibiotics	VA + CAZ		VA + MEM		
**Cerebral function monitoring parameters**					
Analgesics-sedatives	Midazolam + Fentanyl + Rocuronium				
RASS	−5	−5	−4	−2	−2
NIRS-ScO2	55	60	61	65	68
BIS	40	45	51	60	-
TAP (cm/s)	-	72	86	106	100
Perfusion index	-	1.92	1.36	1.04	0.58
**Other parameters**					
Amylopsin (U/L)	69	357	219	72	30
Lipase (U/L)	1,048	1,884	1,317	384	131
cTNI (μg/L)	0.06	1.53	2.64	1.42	0.68
NT-proBNP (pg/ml)	1,874	-	3,589	3,726	1,994
ALT(U/L)	31	460	293	259	215
AST(U/L)	190	1,805	1,027	920	785
TBIL (μmol/L)	5.4	11.2	22.8	23.6	33.6

*ACT, activated clotting time; APTT, activated partial thromboplastin time; AT-III, antithrombin-III; CRP, C-reactive protein; PCT, procalcitonin; VA, Vancomycin; CAZ, Ceftazidime; MEM, meropenem; RASS, Richmond Agitation Sedation Scale; NIRS-ScO2, Cerebral oxygen saturation monitoring by near-infrared spectroscopy; BIS, bispectral index; cTNI, cardiac troponin I; NT-proBNP, N-terminal pro-b-type natriuretic peptide; ALT, alanine aminotransferase; AST, aspartate aminotransferase; TBIL, total bilirubin*.

### Follow-Up and Outcomes

**Figure 2:**
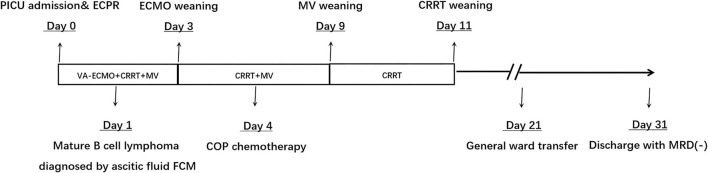
The clinical timeline of the patient.

With ECMO and CRRT treatment, the patient's hyperkalemia was quickly corrected (6 h after the start of CRRT), her cardiac ejection function improved, and her blood lactate gradually fell. Three days after admission, the patient's heart function recovered and ECMO was weaned. Tumor induction chemotherapy was started on day 4, the patient's lung function significantly improved, the ventilator was withdrawn on day 9, and CRRT treatment was discontinued on day 11. Twenty-one days after admission the patient's organ function had recovered well and she was awake and able to communicate normally (Figure 2). Brain function assessment by the Paediatric Cerebral Performance Category got 1 point. Only a small subdural hemorrhage was seen on cranial MRI, and the patient was transferred to the general ward. The patient was discharged 31 days after admission after she completed the first phase of chemotherapy and her bone marrow minimal residual disease (MRD) was negative. In total her ECPR time was 1 h, her ECMO support time was 71 h, her ventilator support time was 190 h, and her CRRT operation time was 247 h. No serious ECMO complications occurred. At the time of discharge, the patient had a good mental function and other organ functions were normal.

## Discussion

The diagnosis and treatment of this patient's case provided us with three important experiences: (1) the use of critical ultrasound technology in the rapid diagnosis and treatment of the cause of shock and critical illness; (2) valuable experience with ECMO management and in the use of craniocerebral ultrasound monitoring of cerebral blood flow; (3) childhood malignant tumors should not be contraindications to the use of ECMO in children, but its use should only be following a comprehensive assessment of their condition.

Shock is a common critical illness in the ICU. ICU care permits the rapid identification of the type of shock and accurate interventions to achieve early recovery and improve long-term prognosis. Critical Care Ultrasound is a problem-oriented, multi-objective integrated dynamic bedside evaluation process under the guidance of critical care medicine theory and the use of ultrasound technology for critically ill patients. It is an important means to determine critical care, especially the direction of hemodynamic therapy and guide fine adjustments (4). We performed a thorough assessment of the patient's heart using the bedside ultrasound, identifying a full lower cavity, enlarged right heart, and a decreased left ventricular ejection fraction. Once the patient's shock type was determined to be cardiogenic, and combined with the appearance of her electrocardiogram, the internal environmental characteristics of high potassium, low calcium, and high phosphorus levels suggest what pathophysiological process was occurring. The contents of the lower abdomen were evaluated using an abdominal ultrasound, and conclusive evidence of a tumor was finally found. The characteristic changes in electrolyte levels caused by tumor lysis syndrome led to arrhythmia, which in turn caused a cardiogenic shock. The characteristics of the course of tumor lysis syndrome lets us know that conventional potassium-lowering methods cannot quickly stabilize the child's internal environment. CRRT is an important medical technique that can quickly correct the body's water, sodium, and electrolyte balance. Therefore, an initial treatment plan of ECMO combined with CRRT was therefore developed to correct the child's internal environment in the shortest possible time. ECMO machine time was minimized, reducing the risk of serious complications that can occur in the patient. The early diagnosis of the cause of this patient's cardiogenic shock using critical ultrasound provided an important basis for the early initiation of ECPR.

In this case, the total CCPR time experienced by the child is close to 1 h, and continuous CCPR time is up to 40 min. The results of a large multi-center cohort study in North America showed that continuous CCPR for more than 21 min without ROSC may worsen neurological prognosis (5). Hence, how to protect the patient's organs, especially her brain, was the focus of ECMO management during her ECMO support period. First, targeted temperature management (TTM) and sedation and analgesia are considered to be important strategies that can improve the neurological prognosis after ECPR (6). Studies have shown that sub-hypothermia treatment can reduce brain oxygen consumption, reduce the production of oxygen free radicals, and significantly improve the prognosis of patients with a traumatic brain injury. International resuscitation guidelines recommend TTM at 32°C−36°C in unconscious patients with out-of-hospital cardiac arrest for at least 24 h (6, 7). Considering the TTM complications such as deterioration of hemodynamics and bleeding, we set the target temperature to 35°C (anal temperature). Combined with deep sedation and analgesia, TTM management lasted for 48 h, and hemodynamic deterioration and chills related to TTM did not occur. In addition, we also adjust the blood pressure and ECMO flow of children by monitoring indicators such as cerebral blood flow, cerebral oxygen, and arterial carbon dioxide partial pressure. We monitored the brain blood flow of the middle cerebral artery (MCA) daily by cranial ultrasound and continuous cerebral oxygen saturation by near-infrared spectroscopy (NIRS). When the ECMO flow is maintained at 80–90 ml/kg/ min and the mean arterial pressure (MAP) > 65 mmHg, the cerebral blood flow TAP can reach more than 70 cm/s, and the cerebral oxygen can also be maintained at 60–80%. It should be noted that CCPR was performed under the monitoring of intraarterial blood pressure (MAP > 60 mmHg), which could ensure a high-quality CPR. There is a greater risk of bleeding and nosocomial infections in patients with cancer when ECMO treatment is performed. At the beginning of treatment, the patient's diagnosis allowed us to predict the possibility of early weaning after a short period of ECMO support. We therefore adopted a more conservative anticoagulation strategy and maintained APTT around 60 s *via* heparin treatment (5–10 U/kg/h). With respect to antibiotics, meropenem and vancomycin were used to prevent infection. Despite this, on the third day of ECMO support, the patient's laboratory reports showed a reduced number of white blood cells, and significantly increased CRP and inflammatory indicators. We used intravenous immunoglobulins to improve the patient's immunity. Furthermore, early weaning is a key factor to preventing nosocomial infections.

The incidence of pediatric malignancies has increased, and they have become the second leading cause of death in children. TLS can cause a malignant arrhythmia due to high potassium and low calcium levels. It is one of the most common emergencies related to malignant tumors. ECMO is rarely used in the management of severe cardiopulmonary failure caused by TLS. Prabhu reported that a 16-month-old child with acute myeloid leukemia developed TLS combined with a respiratory syncytial virus infection after induction chemotherapy, resulting in acute respiratory and circulatory failure. VA-ECMO was used to treat the patient's shock successfully for 16 days (8). Sanford reported an 8-year-old boy with metastatic alveolar rhabdomyosarcoma who developed severe TLS with pulmonary edema and right ventricular failure after chemotherapy. The patient successfully improved after 5 days of VA-ECMO support (9). These two reports are based on tumor lysis and have unique characteristics that suggest that effective ECMO support is possible in children with malignancies. The characteristics of the case in this report are as follows: (1) the child was hospitalized due to sudden cardiogenic shock directly induced by TLS; (2) based on rapid diagnosis of TLS, the treatment of ECMO combined with CRRT was reasonable, ECPR provided a time window for the pathological diagnosis and treatment of the child's tumor; and CRRT enabled the rapid correction of the patient's internal environment, permitted the rapid weaning of ECMO and avoided serious complications. In addition, the child has been in good health history. Therefore, in this case, ECMO+CRRT is an accurate treatment plan.

Tumors are no longer an absolute contraindication to the use of ECMO in critically ill patients. Beyond TLS, ECMO is reported to treat pediatric tumor patients in other critical care situations. First, tumors are space-occupying lesions that can lead to airway and/or cardiac vascular obstruction. ECMO can be used to bridge surgery or chemotherapy. There are many reports about adult cases using ECMO. Bourcier reported five adult patients with respiratory and circulatory failure caused by huge mediastinal tumors at a single center. They had different degrees of obstructive shock when they entered the ICU. After emergency VA-ECMO support, they were diagnosed with cancer and received chemotherapy (10). With respect to pediatrics, Ward reported three successful instances of planned VV-ECMO to bridge surgery in infants with airway obstruction caused by airway tumors. ECMO was weaned soon after surgery without any serious complications (11). Although there have been no rigorous large-scale clinical studies on the topic, a large number of successful cases of ECMO have been reported in cancer patients, which suggests that ECMO may be a rare emerging strategy for the treatment of obstructive shock caused by a primary mediastinal malignancy (12–14).

Second, severe infections can lead to acute respiratory distress syndrome (ARDS) or septic shock, requiring salvage ECMO support during cancer treatment. Children often have long-term immunosuppression and bone marrow hematopoietic insufficiency after chemotherapy or hematopoietic stem cell transplantation (HSCT), which increases their risk of ECMO complications, in particular severe bleeding and infection. Stecher retrospectively analyzed the prognosis of 25 patients with malignant tumors or HSCT who received ECMO for ARDS at a single center. The results showed that all 25 patients had leukopenia/thrombocytopenia due to anti-cancer treatment or underlying disease and that 17 patients (68%) died of ECMO. Among them, four patients had serious bleeding events (15). Bojic et al. retrospectively analyzed the relevant characteristics and results of patients with long-term ECMO managed at a single center (16). The proportion of patients with cancer was significantly lower in the long-term survivor group, and the main complication was an infection. Accordingly, providing ECMO for tumor patients with immunosuppression is still controversial.

In addition, several cases of secondary circulatory crises in patients with pheochromocytoma have been reported along with a summary of cases that used VA-ECMO transitional support therapy (13, 17, 18). Severe respiratory and circulatory failure due to chemotherapy drug-related cardiopulmonary injuries can also require ECMO support. Odish reported six cases of VV-ECMO used for the salvage treatment of lung injuries caused by bleomycin in adult tumor patients, with a cumulative survival rate of 33% (2/6) (19).

In conclusion, there are significant differences in the clinical details of patients with malignancies. There is no standardized protocol for when to provide ECMO. In this case, a girl with primary B-cell lymphoma was previously healthy. The use of ECMO support during an emergency undoubtedly won valuable time for the child. The combined use of ECMO and CRRT considerably shortened the patient's ECMO support time and avoided common complications of ECMO in tumor patients such as bleeding and infection. Decision-makers must balance factors such as disease prognosis, reversibility, the possibility of treatment-related complications, and appropriate use of clinical resources. Under no circumstances should ECMO for potential survivors be rejected simply because of the presence of a malignancy. Centers should continuously improve the management quality of ECMO for this kind of patients, which should improve corresponding results.

## Patient Perspective

I still remember when I was sent to the PICU, I had trouble breathing and chest tightness. I thought I was dying and was terrified. When I woke up I couldn't remember many things, and just felt a little dizzy and in pain. However, the doctor said that my illness had improved a lot and I can return to school again in the future. Now I just need to see my doctor regularly.

## Data Availability Statement

The original contributions presented in the study are included in the article/supplementary material, further inquiries can be directed to the corresponding author/s.

## Ethics Statement

Written informed consent was obtained from the child's legal guardian for the publication of any potentially identifiable images or data included in this article.

## Author Contributions

This work was performed at Shanghai Children's Medical Center, HR contributed to conception and design of the study. ZW organized the database and wrote the first draft of the manuscript. HR and WW has edited and revised the first draft. All authors contributed to manuscript revision, read, and approved the submitted version.

## Funding

This work was supported by a grant from Pudong New Area Science and Technology Development Fund (Grand Number: PKJ2018-Y43).

## Conflict of Interest

The authors declare that the research was conducted in the absence of any commercial or financial relationships that could be construed as a potential conflict of interest.

## Publisher's Note

All claims expressed in this article are solely those of the authors and do not necessarily represent those of their affiliated organizations, or those of the publisher, the editors and the reviewers. Any product that may be evaluated in this article, or claim that may be made by its manufacturer, is not guaranteed or endorsed by the publisher.

## References

[1] Jones GL Will A Jackson GH Webb NJ Rule SH. British Committee for Standards in Guidelines for the management of tumour lysis syndrome in adults and children with haematological malignancies on behalf of the British Committee for Standards in Haematology. Br J Haematol. (2015) 169:661–71. 10.1111/bjh.13403 25876990

[2] Alexander PMA Thiagarajan RR. Pediatric oncology-the final frontier for extracorporeal membrane oxygenation in children? Pediatr Blood Cancer. (2020) 67:e28521. 10.1002/pbc.28521 32785993

[3] Hong J Choi CH. Extracorporeal membrane oxygenation support in patients with hematologic malignancies: to whom and when? Korean J Intern Med. (2017) 32:1116–8. 10.3904/kjim.2016.260 29056036 PMC5668400

[4] Yin WH Wang XT Liu DW Kang Y Chao YG Zhang LN. [A Chinese consensus statement on the clinical application of transesophageal echocardiography for critical care (2019)]. Zhonghua Nei Ke Za Zhi. (2019) 58:869–82. 10.3760/cma.j.issn.0578-1426.2019.12.002 31775449

[5] Reynolds JC Grunau BE Elmer J Rittenberger JC Sawyer KN Kurz MC. Prevalence, natural history, and time-dependent outcomes of a multi-center North American cohort of out-of-hospital cardiac arrest extracorporeal CPR candidates. Resuscitation. (2017) 117:24–31. 10.1016/j.resuscitation.2017.05.024 28552656

[6] Taccone FS Picetti E Vincent JL. High quality targeted temperature management (TTM) after cardiac arrest. Crit Care. (2020) 24:6. 10.1186/s13054-019-2721-1 31907075 PMC6945621

[7] Donnino MW Andersen LW Berg KM Reynolds JC Nolan JP Morley PT. Temperature management after cardiac arrest: an advisory statement by the advanced life support task force of the international liaison committee on resuscitation and the American Heart Association Emergency Cardiovascular Care Committee and the council on cardiopulmonary, critical care, perioperative and resuscitation. Resuscitation. (2016) 98:97–104. 10.1016/j.resuscitation.2015.09.396 26449873

[8] Prabhu AD Mos K Karl TR Anderson B. Extracorporeal life support in the acute management of tumour lysis syndrome. Interact Cardiovasc Thorac Surg. (2012) 15:568–9. 10.1093/icvts/ivs233 22647970 PMC3422945

[9] Sanford E Wolbrink T Mack J Rowe RG. Severe tumor lysis syndrome and acute pulmonary edema requiring extracorporeal membrane oxygenation following initiation of chemotherapy for metastatic alveolar rhabdomyosarcoma. Pediatr Blood Cancer. (2016) 63:928–30. 10.1002/pbc.25879 26713672 PMC5849391

[10] Bourcier S Villie P Nguyen S Hekimian G Demondion P Brechot N. Venoarterial extracorporeal membrane oxygenation support rescue of obstructive shock caused by bulky compressive mediastinal cancer. Am J Respir Crit Care Med. (2020) 202:1181–4. 10.1164/rccm.202001-0193LE 32543883

[11] Huard D Chenouard A Fernandez M Boyer J Guinot A De Napoli-Cocci S. The use of intraoperative peripheral extracorporeal membrane oxygenation in high-risk airways tumor removal procedures in neonates and children: a single-Center case series. ASAIO J. (2021) 67:e176–81. 10.1097/MAT.0000000000001360 33528164

[12] Miles B Durham LA Kurman J Joyce LD Johnstone DW Joyce D. Venovenous extracorporeal membrane oxygenation to facilitate removal of endobronchial tumors. Tex Heart Inst J. (2021) 48:e197111. 10.14503/THIJ-19-7111 34243188 PMC8367282

[13] Wang T Xu Q Jiang X. Successful extracorporeal membrane oxygenation resuscitation of patient with cardiogenic shock induced by phaeochromocytoma crisis mimicking hyperthyroidism: a case report. Open Life Sci. (2021) 16:746–51. 10.1515/biol-2021-0073 34316515 PMC8285988

[14] Zhang Y Luo M Wang B Qin Z Zhou R. Perioperative, protective use of extracorporeal membrane oxygenation in complex thoracic surgery. Perfusion. (2021). 10.1177/02676591211011044. [Epub ahead of print]. 33908283

[15] Stecher SS Beyer G Goni E Tischer J Herold T Schulz C. Extracorporeal membrane oxygenation in predominantly leuco- and thrombocytopenic haematologic/oncologic patients with acute respiratory distress syndrome - a single-centre experience. Oncol Res Treat. (2018) 41:539–43. 10.1159/000489718 30114706

[16] Bojic A Schellongowski P Robak O Hermann A Buchtele N Nagler B. Long-term respiratory extracorporeal membrane oxygenation and prognosis: a retrospective analysis. ASAIO J. (2021) 67:345–52. 10.1097/MAT.0000000000001225 33627611

[17] Martin-Villen L Corcia-Palomo Y Escalona-Rodriguez S Roldan-Reina A Acosta-Delgado D Martin-Bermudez R. Extracorporeal membrane oxygenation support in a patient with pheochromocytoma stress myocardyopathy. Med Intensiva. (2018) 42:566–8. 10.1016/j.medine.2018.04.008 29764675

[18] Min D. Catastrophic catecholamine-induced cardiomyopathy rescued by extracorporeal membrane oxygenation in recurrent malignant pheochromocytoma. Yeungnam Univ J Med. (2019) 36:254–9. 10.12701/yujm.2019.00213 31620641 PMC6784645

[19] Odish MF McGuire WC Thistlethwaite P Crotty Alexander LE. Bleomycin-induced lung injury treated with venovenous extracorporeal membrane oxygenation (ECMO) and ultra-protective ventilator settings. BMJ Case Rep. (2020) 13:e236474. 10.1136/bcr-2020-236474 33229479 PMC7684647

